# Forest dynamics

**DOI:** 10.12688/f1000research.7412.1

**Published:** 2016-02-17

**Authors:** Lee Frelich

**Affiliations:** 1 University of Minnesota Center for Forest Ecology, St. Paul, Minnesota, 55108, USA

**Keywords:** Forest dynamics, Succession, neighborhood theories, neighborhood effect, disturbance dynamics

## Abstract

Forest dynamics encompass changes in stand structure, species composition, and species interactions with disturbance and environment over a range of spatial and temporal scales. For convenience, spatial scale is defined as individual tree, neighborhood, stand, and landscape. Whether a given canopy-leveling disturbance will initiate a sequence of development in structure with little change in composition or initiate an episode of succession depends on a match or mismatch, respectively, with traits of the dominant tree species that allow the species to survive disturbance. When these match, certain species-disturbance type combinations lock in a pattern of stand and landscape dynamics that can persist for several generations of trees; thus, dominant tree species regulate, as well as respond to, disturbance. A complex interaction among tree species, neighborhood effects, disturbance type and severity, landform, and soils determines how stands of differing composition form and the mosaic of stands that compose the landscape. Neighborhood effects (e.g., serotinous seed rain, sprouting, shading, leaf-litter chemistry, and leaf-litter physical properties) operate at small spatial extents of the individual tree and its neighbors but play a central role in forest dynamics by contributing to patch formation at stand scales and dynamics of the entire landscape. Dominance by tree species with neutral to negative neighborhood effects leads to unstable landscape dynamics in disturbance-prone regions, wherein most stands are undergoing succession; stability can only occur under very low-severity disturbance regimes. Dominance by species with positive effects leads to stable landscape dynamics wherein only a small proportion of stands undergo succession at any one time. Positive neighborhood effects are common in temperate and boreal zones, whereas negative effects are more common in tropical climates. Landscapes with positive dynamics have alternate categories of dynamics stabilized by high-severity and low-severity disturbance regimes. Contrary to prevailing ecological theory, systems with positive neighborhood effects can have similar levels of compositional stability across tree, stand, and landscape scales. Neighborhood effect theory can help explain responses of landscapes to large-scale land clearing and novel effects brought on by factors such as invasive species and deer overabundance.

## Introduction

Forest dynamics encompass changes in stand structure, species composition, species interactions with disturbance type and severity, and disturbance interactions with landform, over a range of spatial and temporal scales ^1– 4^. This article covers forest dynamics in the context of landscape ecology and patch dynamics theory. Forest dynamics as related to biological legacies, land use, herbivory, tree diseases, carbon storage, biogeochemistry, forest health, and climate change are covered elsewhere.

This review is rooted in classic concepts of landscape ecology, including hierarchical patch dynamics ^5, 6^, emergent properties at large spatial extents from interactions among individuals ^7^, resilience, and increasing stability/slower dynamics at landscape than at stand scales ^5, 8, 9^, as well as the forestry concepts of disturbance severity, stand development, and succession ^10^. However, here neighborhood effects are added to the traditional tree-stand-landscape hierarchy, and stability is examined from the viewpoint of species composition rather than age-class distribution of disturbance patches. This changes the properties of landscape mosaics; four categories of landscape dynamics emerge from a neighborhood effect point of view and they include some seemingly strange elements, such as high stability at small and large spatial extents, and two different types of shifting mosaic steady states. In addition, neighborhood effect theory shows how species not only respond to disturbance but also sometimes regulate disturbance.

The article starts with a review of forest development and succession, which sets the stage for discussions of species-disturbance interactions, integration of neighborhood effects into cross-scale dynamics, and categories of landscape dynamics. Throughout the article, spatial scales and disturbance severity, as defined in 3, are used. These are individual tree (approximately 0.01 ha), stand, a spatially contiguous collection of trees with similar composition and age structure (0.1 to 10 ha), and landscape, a collection of adjacent stands in the range of 1000 to 100,000 ha. Disturbance severity will have three general categories: low-severity disturbances causing minor mortality in the canopy or understory, or both (e.g., treefall gaps), moderate-severity disturbances causing major mortality of either the overstory or understory (e.g., canopy-leveling wind and moderately severe surface fire), and high-severity disturbances causing major mortality of the overstory and understory (e.g., canopy-leveling wind followed by fire, or high-intensity crown fire). Cumulative disturbance severity includes the additive impacts of multiple disturbances over time that may influence a stand.

## Development and succession

Stand development is directional change in structure over time, whereas succession is directional change in species composition, where early-successional species are replaced by late-successional species ^3^. A sequence of developmental or successional stages can occur by themselves or together. A stand-leveling event such as high-intensity fire, windthrow, or clearcut logging may reset development or succession (or both) to the early stages.

Development occurs in four stages ^3, 10^: (1) stand initiation, lasting from the time of canopy disturbance until a new canopy of young trees forms. These are often even-aged stands, or at least even-aged from time of release in cases where advanced regeneration was present. On sites with severe environments, recruitment of new trees may take decades, so that new stands may be unevenly aged by the time of canopy closure. (2) Stem exclusion, lasting from the time of canopy closure through the density-dependent process of self-thinning. (3) Demographic transition (also called understory reinitiation ^10^), begins when tree crowns and resulting gaps formed when trees die are large enough so that trees adjacent to a gap cannot fill them by lateral crown expansion; new cohorts of trees can grow into the overstory. (4) Multi-aged stage (also called old growth ^10^), reached when stage 3 has progressed to the point where only a few individuals from the original even-aged cohort remain, and the canopy has trees of widely varying ages; this stage lasts until another disturbance resets stand development. If the initiation stage was dominated by shade-intolerant, early-successional species, then secondary succession driven by tree species of moderate to high shade tolerance entering the stand may occur along with stand development. However, stand development sequences entirely dominated by late-successional, shade-tolerant species also occur ^11^.

During stages 3 and 4 of development, gaps may be filled with small even-aged cohorts of saplings that themselves undergo self-thinning, just like the entire stand did during stages 1 and 2, so that the development process is mirrored in many places at smaller spatial extents. Gap sizes also commonly change from mostly small (50 to 100 m ^2^) in stage 2 and early stage 3 to a wide variety of sizes in late stage 3 and stage 4 (50 to 400 m ^2^); the larger gaps present in these later stages of development lead to opportunities for mid-tolerant species (e.g., *Fraxinus*, *Pinus*, *Prunus*, and *Quercus* in temperate forests) to maintain or increase their presence in the forest ^12, 13^. The light level of the entire stand may be higher in old multi-aged stands, with gaps of widely ranging age and size because side light from numerous gaps can penetrate into the forest around gap edges, so there is no ‘interior zone’ not impacted by gaps ^14^. In addition, partial disturbances that remove 20% to 50% of the canopy, but do not reset the stand to the initiation stage of development, have a large influence on forest dynamics in areas with wind-and-gap types of regimes, and stands hit by such disturbances have relatively high light levels for a few decades after disturbance ^15^.

Traits that determine how a given tree species survives disturbance interact with disturbance type and severity to determine whether a given disturbance will perpetuate the existing species composition or initiate an episode of succession ^16, 17^. When disturbance characteristics match survival mechanisms of the dominant tree species then regeneration to pre-disturbance species occurs with little change in composition and development is reset without resetting succession. A mismatch between tree survival mechanisms and disturbance leads to reduction in dominance of pre-disturbance species. Some of the trait-disturbance matching syndromes include (1) species with canopy-stored seed banks, which have several seasons of seeds on hand at any given time, survive disturbance as seeds, and are adapted to high-intensity crown fires on dry sites ^1^, (2) shade-tolerant, late-successional species growing on mesic sites with a seedling/sapling bank in the understory that can respond to overstory wind disturbance ^18, 19^, and (3) shade-intolerant or mid-tolerant species with thick bark and foliage held high above the ground, which survive as mature trees adapted to surface fire regimes ^20^. Type 1 forests are maintained at an early-successional stage by fire, and lack of fire or a novel disturbance type (e.g., wind) may never activate the seed bank, leading to recruitment failure and succession to another species. A mismatch for type 2 could be a severe disturbance killing almost all mature trees, seedlings, and seeds. Type 3 forests are usually maintained at a mid-successional stage by fires and could have mismatches caused by a fire that is more severe than normal, killing the mature trees and allowing an earlier-successional species to take over, or lack of fire, allowing late-successional species to dominate ^21^.

Sprouting from stumps, root systems, or trunks with broken crowns is a type of survival that is often underappreciated and can allow adaptation to various disturbance types. Crown sprouting, such as occurs in tropical forests after hurricane damage ^22^, is a form of mature-tree persistence that is not common in temperate and boreal forests, although the author has noted that yellow birch ( *Betula alleghaniensis*) and cottonwood ( *Populus deltoides*) are exceptions. Stump and root sprouts are a common form of persistence in aspen ( *Populus* spp.), birch ( *Betula* spp.), and oak ( *Quercu*s spp.) species in temperate and boreal forests ^1, 23^.

Successional status in boreal species can be quite confusing; owing to the low richness of the regenerating species pool, one species can fill more than one successional role. For example, paper birch ( *Betula papyrifera*) and black spruce ( *Picea mariana*) in North America can dominate early post-fire stands as well as old multi-aged stands ^24, 25^. This contrasts with progressively higher species turnover across successional gradients as mean annual temperature increases from temperate to tropical forests.

Structural complexity of dead wood and microtopography can influence regeneration dynamics. These biological legacies that persist through, or are formed by, disturbance can have large effects on regeneration dynamics—forests can be ‘born complex’ ^26^. Variation in location of large coarse woody debris and pit-and-mound topography can have differential influence on growth and success among regenerating species ^27^, possibly allowing seedlings of some late-successional species to survive after very large, high-severity disturbances.

## Role of tree species and landform in disturbance

Tree species have a surprising ability to regulate disturbance frequency and effects by producing fuel structures at stand and landscape scales that promote high or low fire intensity and rate of spread. Tree susceptibility to wind is a function of wood strength, wood flexibility, tree growth form (tall and thin: susceptible; low center of gravity: less susceptible), and ability to streamline in the wind or to shed branches to reduce pressure caused by wind. Species resistant to wind tend to be late-successional, with some exceptions such as bur oak ( *Quercus macrocarpa*), and arrive at their resistance by various combinations of traits ^28^.

Although it is inevitable that any quasi-equilibrium that may exist at the landscape scale will be disrupted at some point, species have a tendency to ‘lock in’ a certain disturbance regime on a certain landform for multiple tree generations—during this time, disturbance and tree traits are in sync—or perhaps out of sync only for a small proportion of stands across the landscape ^29^. Examples ( Table 1) include ‘fire pines’ such as jack pine ( *Pinus banksiana*) in the North American boreal forest ^30, 31^, growing on dry sites at high canopy densities with repeated high-intensity crown fires at intervals greater than necessary to reach seed-bearing age but shorter than needed for later-successional species to invade. A second example includes eastern hemlock ( *Tsuga canadensis*) and sugar maple ( *Acer saccharum*) that grow on mesic sites not conducive to fire but that reproduce well after treefall events caused by wind at a variety of spatial extents ^32^. A third example is white pine ( *Pinus strobus*) growing on dry-mesic sites with thick bark and low canopy bulk density that favor surface fires in mature stands.

**Table 1.** Tree species trait and disturbance interactions.

	Forest type-dominant tree species		
	Jack pine	White pine	Sugar maple
Survive disturbance as	Seed (serotinous cones)	Mature trees (thick bark)	Seedlings and saplings (shade-tolerant)
Optimal disturbance type and severity	High-intensity crown fire	Moderate-severity surface fire	Low-severity treefall by wind
Successional status	Early	Middle	Late
Typical site types	Dry, sand, or shallow to bedrock	Dry-mesic, sandy loam	Mesic, loam, or silt loam
Cause of succession/mismatch	Low cumulative severity of disturbance due to lack of fire	Lack of fire or high-intensity fire	Blowdown-fire combination

The examples show species trait, disturbance, and site type combinations that work together to lock in dominance by a given species. The last row also shows disturbance types that are a mismatch and can cause replacement of the dominant species by setting back or advancing succession.

**Figure 1.**
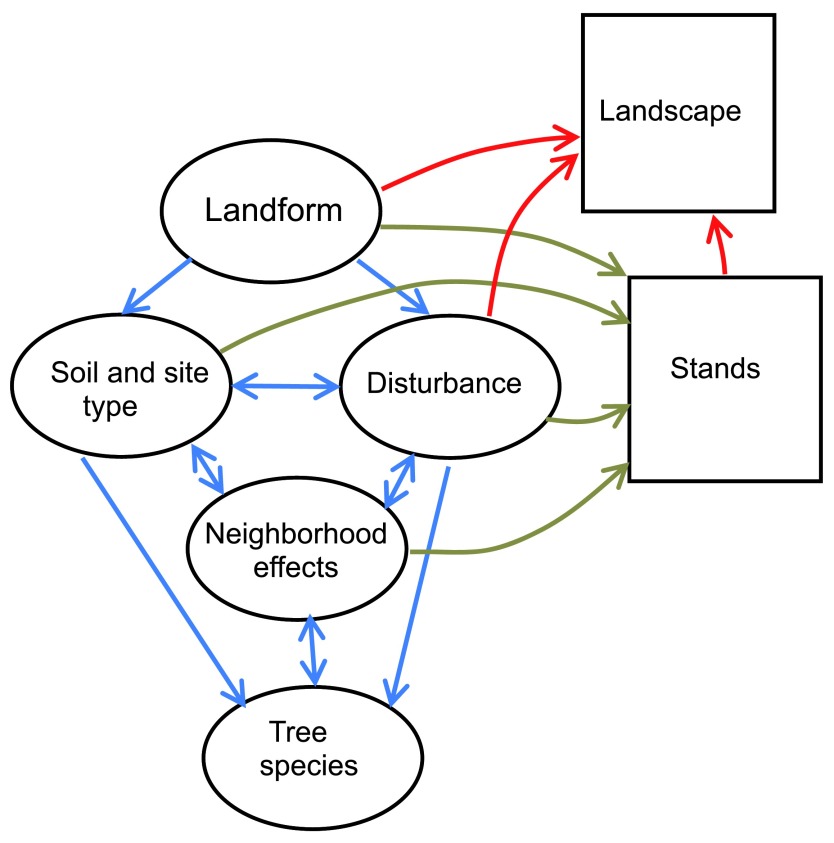
Processes of stand and landscape formation. Note the central role of neighborhood effects in mediating the relationships among tree species, disturbance, and soil characteristics (blue arrows). Via neighborhood effects, tree species can indirectly influence three of the four factors in stand formation (brown arrows). Stands, combined with landform and disturbance, in turn form the landscape (red arrows).

Species mosaics can form because of landform-disturbance-tree species interactions, even within one large disturbance patch ( Figure 1). This could be caused by spatial variation in disturbance severity associated with landform and also by the fact that different species adapted to the same disturbance type and severity could have differential success on various soil types present within the larger disturbance ^33^. Superimposed on this is additional patch variability caused by the fact that succession is a spatial process; waves of disturbance-sensitive species can move out from surviving populations at disturbance edges and refuges within disturbance perimeters ^4^. Thus, the spatial distribution of landform features at a ‘meso-scale’ (larger than stand but smaller than landscape scale) determines the magnitude of the effects of disturbance size, and landscape resilience to large disturbances ^2^. For fire-sensitive species that can survive fires in swamps or lakeshores, the distribution of such refuge areas across the landscape could cause large variance in the time taken for fire-sensitive, late-successional species to reach stands across the landscape. If one such refuge exists on every 10 ha, for example, then late-successional, fire-sensitive balsam fir ( *Abies balsamea*) could reach the understory of most jack pine forests within several decades, even within a large 100,000-ha fire perimeter. If no such refuges exist, then succession in the middle of a large burn may not take place because of simple lack of availability of late-successional species. In general, refuges from disturbance are frequent on forested landscapes, and overall disturbance size matters much less than one would think by looking at fire perimeter maps. Landscape heterogeneity can provide sites with coarse sandy or rocky soils that serve as permanent seed sources for early-successional species on a landscape otherwise dominated by late-successional species (e.g., white pine in a hemlock-hardwood landscape in northern Wisconsin) ^32^. In addition, landform heterogeneity can divide the landscape into patches (or landscape subsections) with different disturbance regimes, levels of connectivity with other nearby occurrences of the same forest type, and different types of dynamics. In mountainous regions, elevation, aspect, and shape of valleys and ridges create patches that dry out more or less frequently, influencing the frequency, intensity, and direction of movement of fire and wind disturbance, and restricting species movements in response to disturbance. Repeating patterns of lakes, swamps, and rocky ridges can have similar effects in the vast, relatively flat boreal forests of central North America.

## Integration of dynamics across scales

The hierarchical tree, stand, and landscape concept has been commonly employed to understand forest dynamics ^3^. At the landscape scale, one can examine the proportion of stands in various stages of succession and development as a result of disturbance history ^1^, as well as proportions of the landscape that may represent different successional pathways due to site type differences—a given landscape is likely to have different successional systems on soils with different water-holding capacity and nutrient status or on subsections of the landscape with different disturbance regimes as mentioned above ^33, 34^. At the stand scale, one can examine changes in tree size distributions and species due to development and succession ^21, 35^.

A fourth scale, neighborhood (individual trees plus the surrounding neighbors), is also needed to explain forest dynamics. Neighborhood effects are an additional mechanism of patch/stand formation. Trees alter the success of their own regeneration under their own canopy and those of adjacent trees through leaf-litter chemistry and physical characteristics, degree of shade and season(s) of shade cast by a given species, seed rain, sprouting, and disease dynamics ^36– 38^. These neighborhood effects can be positive (conspecific regeneration favored), neutral (no effects on abundance of conspecific regeneration), or negative. Neighborhood effects can be mediated by overstory-understory effects within mid- to late-successional forests or disturbance-activated effects such as seed rain from serotinous seeds or sprouting after fire in early-successional forests. It appears that tropical systems have many species with negative neighborhood effects due to disease impacts on seedlings ^39^. These disease feedbacks are uncommon in the temperate zone, although red pine ( *Pinus resinosa*) is an exception ^40^. In temperate and boreal biomes, there are many species whose seedlings do better near conspecific adults, although neutral species are also common ^41^.

Why are neighborhood effects such an important addition to the traditional tree, stand, and landscape scales? These effects play a central role in mediating the interaction among tree species, soils, and disturbance; they allow tree species to influence three of the four main stand-forming factors ( Figure 1). Positive neighborhood effects can cause stands of differing species composition to form on uniform soils in late-successional forests (e.g., hemlock versus sugar maple) and can also cause memory of patch composition through a severe disturbance, as occurs with aspen (root sprouts) versus jack pine (seed rain) patches returning after severe fire ^3^. In effect, strong positive neighborhood effects make the dispersal distance for the pre-disturbance species to recolonize the disturbed area effectively zero—as either seedlings or seeds are present immediately after disturbance throughout a disturbance patch. With positive neighborhood effects, disturbance patches can cross stands dominated by different species, so their boundaries are independent of patch boundaries created by neighborhood effects. Furthermore, patches dominated by different species with positive neighborhood effects act together to influence dynamics of disturbance at larger spatial extents (details below). Thus, neighborhood effects operate at the scale of adjacent trees (0.01 to 0.05 ha) but can create much bigger patches (1 to 10 ha or more) and influence landscape-scale dynamics ( Figure 1).

Without neighborhood effects, all gaps would have random replacement among the species present, and no patches of different species could form on the same soil type independently of disturbance. Neighborhood effects can and do allow coexistence among species with very similar environmental tolerances, via the division of the initially uniform environment into spatially separate patches. Thus, the neighborhood scale is needed to understand patch dynamics, even for locations on uniform soils or within large, high-severity disturbance patches.

## Positive and neutral/negative neighborhood theories of forest dynamics

The neighborhood effect theory of forest dynamics was proposed by 3, 41. This theory used the cusp catastrophe model ^42– 44^ to predict that both continuous and threshold changes in species composition can occur over time. Forests dominated by species with positive neighborhood effects (e.g., serotinous seed rain after fire or a conspecific seedling bank after wind) have a better memory of pre-disturbance composition at neighborhood and stand scales, over a wider range of disturbance severities, than forests dominated by neutral or negative neighborhood species. In essence, predicting forest dynamics without taking neighborhood effects into account amounts to assuming that neighborhood effects are neutral in all cases, and this would therefore be a neutral theory of forest dynamics. Neighborhood effect theory is a more general case that encompasses negative/neutral and positive dynamics.

The theory also predicted that alternate states of composition (early and late successional) can occur after disturbances of similar severity on landscapes dominated by tree species with positive neighborhood effects, depending on the history of composition. A contrasting continuous response was predicted for forests with neutral neighborhood effects. These predictions were shown to be valid in several case studies ^41^.

**Figure 2.**
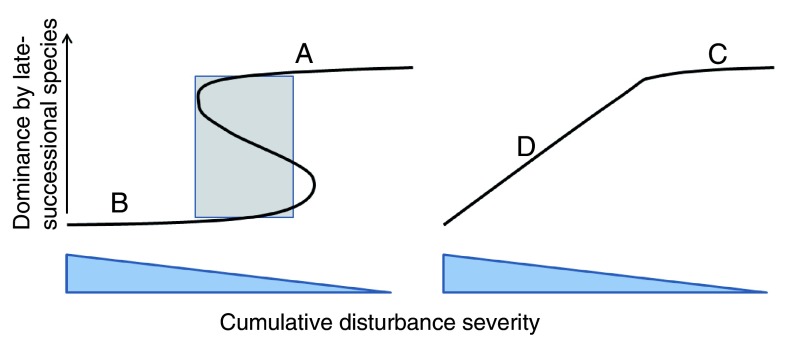
Response surface to disturbance severity and species composition for forests dominated by trees with positive neighborhood effects (left) and neutral to negative neighborhood effects (right). For positive neighborhood effects, categories A and B represent late- and early-successional landscapes stabilized by low- to moderate-severity wind disturbance regimes and moderate- to high-severity fire regimes, respectively. Some stands may make temporary excursions to the opposite category, but most stands hover on or near the ‘A’ or ‘B’ parts of the response surface. Note that the area of coexistence of early- and late-successional types at moderate disturbance severities depends on history for a given stand (shaded area, left) and that the range of severities that will allow persistence is quite large for A and B. For neutral to negative neighborhood effects, any change in severity over time for D will cause a change in composition as stands slide up and down the response surface. Composition of stands in C can be changed with much less effort than for stands in A; therefore, very stable, low-severity disturbance regimes are required over time to allow C dynamics to persist.

The third major prediction of the theory was the existence of four landscape dynamic categories that emerged from a scaled-up impact of neighborhood effects ( Figure 2). The category for a given landscape is related to the portion of the response surface where most of the stands hover, or the portion of the surface to which they are attracted, given the dominant species-disturbance interactions. Category A and B landscapes have high stability across all spatial scales, tree, stand, and landscape because of positive overstory-understory (A) or disturbance-activated neighborhood effects (B). Although prevailing ecological theory recognizes that landscape dynamics can be stabilized by low- or high-frequency disturbance, it does not allow for the substantial degree of stability at smaller spatial extents (e.g., tree and stand) that is allowed for by positive neighborhood effect theory ^3, 9^. Other characteristics are mostly opposite; A has mostly late-successional stands and is stabilized by low- to moderate-severity disturbance regimes such as gap dynamics, stand-leveling windthrow, and surface fires, whereas B has mostly early-successional stands and is stabilized by moderate- to high-severity disturbance. Severe disturbance beyond the threshold resets succession in A, whereas in B a lack of disturbance needed to maintain early-successional status allows succession to occur. Note that both A and B landscapes can have mosaics of stands with differing species composition if more than one species with strong positive neighborhood effects is present.

Category C landscapes have neutral to negative neighborhood effects and low- to moderate-severity disturbance regimes. Tree species can replace each other after small, low-severity disturbances like gap formation, and there is no memory of species composition at individual tree and neighborhood scales. There can be stability at stand to landscape scales for C landscapes, although in temperate and boreal forests, C-type dynamics are very likely to be ephemeral and wiped out by large-scale disturbance or replaced by species with A-type dynamics. However, tropical rain forests, with their negative density-dependent relationships between mature trees and seedling density ^39^ and low chance of fire or other severe disturbance (in the absence of human land-clearing), can likely persist as category C landscapes for centuries, or as long as the climate is stable.

Category D landscapes are unstable at all spatial scales, as species with neutral neighborhood effects allow composition to change continuously with disturbance severity. A sequence of disturbances of equal severity and uniform temporal spacing would be needed to keep composition the same—an unlikely scenario except perhaps for frequently burned savannas. These are often forests with mixed-severity fire regimes in forest types like Douglas fir ( *Pseudotsuga menziesii*), white pine, and red oak ( *Quercus rubra*), with examples described in 14, 18, 45.

Note that the traditional steady-state mosaic concept in forest dynamics, defined by 46 as “... an array of irregular patches composed of vegetation of different ages”, did not specify stable or unstable composition at individual tree and neighborhood spatial scales and left the concept vague with respect to steady state within a stand or among stands across a landscape. Neighborhood effect theory divides the steady-state mosaic with gap dynamics as the main disturbance regime into two types of dynamics, A and C, depending on whether tree gap-forming species are replaced with conspecifics or a random assortment of species.

## New implications and predictions of neighborhood effect theory

Neighborhood theory predicts the existence of disturbance-mediated accelerated succession ^47^ at stand and landscape scales. If positive disturbance-activated neighborhood effects maintained by fire disappear because of chance absence or exclusion of fire, then late-successional species can enter stands in the understory. A low-severity disturbance regime of gap dynamics or a sudden moderate-severity canopy-leveling windstorm could cause slow or fast transition, respectively, to late-successional species, accompanied by transition from B or D dynamics to A dynamics.

The theory predicts limitations and (possibly unanticipated) exaggerations of management effects at stand to landscape scales. Landscape dynamics category can be changed by human actions. A very severe disturbance at the landscape scale can override the pre-disturbance memory of the entire landscape and cause a switch from A to B landscape dynamics and initiate widespread succession (as happened when European settlers cleared and burned mesic forest landscapes occupied by sugar maple and hemlock in the Lake States of Michigan, Wisconsin, and Minnesota, USA, in the late 1800s), or the artificial exclusion of severe fire can cause the opposite switch, from B to A (accelerated succession mentioned above or fire suppression in some pine forests ^21^).

The theory predicts how certain human-induced environmental changes will affect succession and development. Novel ‘disturbances’ and processes may be able to knock out positive neighborhood effects, leading to changed dynamics. For example, the European earthworm invasion is resulting in loss of the organic horizon in North American forests ^48^, possibly removing neighborhood effects that operate through leaf-litter effects, while overabundant deer could eliminate the seedling layer ^49^ that is crucial to positive overstory-understory effects. Either of these could destabilize the hemlock and maple mosaics common in eastern North America that were maintained by positive neighborhood effects.

## Conclusions

Tree species have a surprising level of control over disturbance dynamics because some tree species can ‘lock in’ disturbance and species interactions on certain landforms to create periods of stability in composition at the landscape scale ^41^, which may end when a large infrequent disturbance occurs ^17^. However, the basic tree-trait and survival mechanism interactions with disturbance as an arbiter of whether the pre-disturbance species will persist after disturbance, or whether succession will be reset, need to be reassessed in light of the cross-scale impacts of neighborhood effects and landform effects. Landform and soil-type effects can create mosaics of stand types within single disturbance patches, whereas neighborhood effects can cause different stand types to separate even within one uniform soil type, and neighborhood effects also cause interactions of groups of positive or neutral species with disturbance to form different categories of landscape dynamics. Thus, the tree-stand-landscape hierarchy is not as straightforward as it may first appear because of the complex cross-linkages among levels and ability of tree species to extend their influence very strongly to stand and landscape scales via neighborhood effects that modify disturbance dynamics ( Figure 1). From an ecological process and disturbance dynamics perspective, the hierarchy does not nest as neatly as it may from a spatial patch dynamics perspective. A complete theory integrating all of the elements in Figure 1 awaits development.

Unfortunately, not enough is known to parameterize the neighborhood effect cusp catastrophe model and predict the actual threshold disturbance severity for a state change, and this is due to difficulties in developing universal measures of disturbance severity and neighborhood effect strength. For now, the theory can be used in a semi-quantitative sense with disturbance severity and neighborhood effect categories. A breakthrough is needed in understanding of disturbance severity and how to measure it, as well as disturbance severity versus disturbance type effects, which are frequently confounded.
